# Ketamine: Neuroprotective or Neurotoxic?

**DOI:** 10.3389/fnins.2021.672526

**Published:** 2021-09-10

**Authors:** Divya Choudhury, Anita E. Autry, Kimberley F. Tolias, Vaishnav Krishnan

**Affiliations:** ^1^Department of BioSciences, Rice University, Houston, TX, United States; ^2^Dominick P. Purpura Department of Neuroscience, Albert Einstein College of Medicine, Bronx, NY, United States; ^3^Department of Psychiatry and Behavioral Sciences, Albert Einstein College of Medicine, Bronx, NY, United States; ^4^Department of Neuroscience, Baylor College of Medicine, Houston, TX, United States; ^5^Department of Neurology, Baylor College of Medicine, Houston, TX, United States; ^6^Department of Psychiatry and Behavioral Sciences, Baylor College of Medicine, Houston, TX, United States

**Keywords:** ketamine mechanism, neuroprotection, ketamine-induced neurotoxicity, BDNF, NMDA receptor, AMPA receptor, antidepressant

## Abstract

Ketamine, a non-competitive *N-*methyl-D-aspartate receptor (NMDAR) antagonist, has been employed clinically as an intravenous anesthetic since the 1970s. More recently, ketamine has received attention for its rapid antidepressant effects and is actively being explored as a treatment for a wide range of neuropsychiatric syndromes. In model systems, ketamine appears to display a combination of neurotoxic and neuroprotective properties that are context dependent. At anesthetic doses applied during neurodevelopmental windows, ketamine contributes to inflammation, autophagy, apoptosis, and enhances levels of reactive oxygen species. At the same time, subanesthetic dose ketamine is a powerful activator of multiple parallel neurotrophic signaling cascades with neuroprotective actions that are not always NMDAR-dependent. Here, we summarize results from an array of preclinical studies that highlight a complex landscape of intracellular signaling pathways modulated by ketamine and juxtapose the somewhat contrasting neuroprotective and neurotoxic features of this drug.

## Introduction

Over the past several years, ketamine has garnered significant interest as a novel therapeutic within the research and lay/media communities for its efficacy as a rapidly acting antidepressant. In March 2019, the United States Food and Drug Administration approved esketamine nasal spray as an adjunct therapeutic for treatment-resistant depression (Duman et al., 2016; Daly et al., 2019; Kim et al., 2019). As ketamine infusion protocols gradually become integrated into mainstream psychiatry practices (Sanacora et al., 2017), a large body of preclinical research, encompassing a range of model systems, has shed light on the complex pharmacodynamic landscape of ketamine, which extends well beyond *N*-methyl-D-aspartate receptor (NMDAR) antagonism. A growing body of research seeks to identify one or more biologically active metabolites of ketamine (Zanos et al., 2016) or agents with similar pharmacodynamic properties (Kang et al., 2017) that may avoid psychotomimetic and cardiovascular side effects (Zanos et al., 2016). As clinical protocols for repetitive ketamine treatments continue to evolve, delineating the long-term safety profile of ketamine administration remains a work in progress. In this review, we summarize a body of basic science literature to provide a forecast about ketamine’s potential as a neuroprotective agent (*defined* as a treatment that promotes glial/neuronal health during adversity), as well as clarify ketamine’s cellular actions that may cause it to function as a neurotoxic agent (resulting in adverse neuronal/glial health or cell death).

### On the History of Ketamine

Ketamine was first synthesized in 1962 as a shorter acting analog of phencyclidine with less intense postoperative delirium (Li and Vlisides, 2016), and was approved for use in humans as an anesthetic in the United States by 1970 (Dong and Anand, 2013; Mion, 2017). Ketamine began to be abused as a dissociative psychotomimetic in the late 1970s, primarily by soldiers in the Vietnam War (where it was widely used as a battle-field analgesic/anesthetic), and on the East Coast of the United States (Mion, 2017). The Drug Enforcement Agency classifies ketamine as a Schedule III drug because of its low to moderate potential for dependence (Lopez and Tadi, 2020). Ketamine’s anesthetic effects require infusions at a rate of ∼1 mg/kg/hr, and its low cost has made it useful for surgeries in low-resource settings, in pediatric age groups, and in those vulnerable to hypotension. It has also been applied as an adjunct treatment for chronic pain (∼0.1 mg/kg/hr), including for patients with opioid-induced hyperalgesia. The ability of *sub*anesthetic ketamine (0.5 mg/kg over ∼40 min) to serve as a fast-acting antidepressant in treatment-resistant depression was demonstrated first in a clinical trial published in 2000 (Berman et al., 2000; Abdallah et al., 2018). More widespread and routine clinical use has been limited by ketamine’s side effects and its abuse potential (Pribish et al., 2020). Nevertheless, its antidepressant properties have since been studied extensively, and more recent research suggests that the neuroprotective effects of subanesthetic dose ketamine infusions may provide a novel therapeutic avenue for acute neuronal injury, neurodegenerative disorders, and other neuropsychiatric disorders (Bell, 2017; Pribish et al., 2020).

## On the Cell Membrane Pharmacology of Ketamine

Ketamine is a non-competitive antagonist of NMDARs. NMDARs are Ca^2+^-permeable ion channels that play critical roles in synaptic function, plasticity, learning, and memory as well as excitotoxicity and nervous system injury and disease (Cull-Candy et al., 2001; Zhou and Sheng, 2013). NMDARs function as heterotetramers comprised of two obligatory GluN1 subunits in combination with two GluN2 (A-D) and/or GluN3 (A,B) subunits, each with distinct biophysical, pharmacological, and signaling properties (Cull-Candy et al., 2001). NMDAR activation requires both the binding of the excitatory neurotransmitter glutamate (with co-agonist glycine or D-serine) and depolarization, which relieves a voltage-dependent magnesium (Mg^2+^) block to allow cation entry. Ketamine inhibits NMDARs at sub-micromolar concentrations, and its inhibitory actions are enhanced by Mg^2+^ in a subunit specific manner (Kotermanski and Johnson, 2009). Stereoisomers and metabolites of ketamine [such as esketamine or (2S,6S)-hydroxynorketamine] also inhibit NMDARs to varying degrees. Multiple lines of evidence across a range of preclinical models indicate that NMDAR antagonism plays a central role in ketamine’s neuroprotective effects, particularly as it relates to glutamate-mediated excitotoxicity and downstream dysregulation of calcium homeostasis following status epilepticus (SE) or traumatic brain injury with administered doses ranging from 5 to 45 mg/kg (Yang et al., 2016; Fujikawa, 2019; Pribish et al., 2020). For example, one study showed that ketamine (45 mg/kg) and midazolam (4.5 mg/kg) were equivalent in their ability to abort SE and reduce its long-term effects in adult rats (Niquet et al., 2017). Another study looking at neuroprotection against SE-induced brain damage during developmental periods demonstrated that blockade of GluN2B-containing NMDARs by CI-1041 (10 mg/kg) and ketamine (25 mg/kg) substantially reduced SE-induced neurodegeneration and microglial recruitment/activation (Loss et al., 2019). The neuroprotective effects achieved by ketamine’s NMDA antagonism may extend to other conditions associated with a loss of blood-brain barrier integrity (such as depression, schizophrenia, and high grade glial tumors), negating the potentially deleterious excitotoxic effects of plasma-circulating glycine and glutamate (Saija et al., 1989; Sumiyoshi et al., 2004; Madeira et al., 2018; Dudek et al., 2020).

Insights into the neuroprotective features of ketamine may be extrapolated from a rich and recent rodent literature on the mechanisms of ketamine’s rapid antidepressant actions, which have been linked to increases in hippocampal neurogenesis (Yamada and Jinno, 2019), enhanced synaptogenesis (Deyama and Duman, 2020), and synaptic connectivity (Abdallah et al., 2018). NMDAR antagonism is central to ketamine’s therapeutic potential and extends to its antidepressant-like properties. Autry et al. (2011) demonstrated that a single ketamine injection (3 mg/kg) produced a rapid antidepressant effect on the forced swim test (FST) that was similar to (and more long lasting than) the NMDA antagonist MK-801 (0.1 mg/kg), reproducing earlier results by Maeng et al. (2008) (2.5 mg/kg). Similarly, a selective blockade of GluN2B-containing NMDARs produced rapid antidepressant-like effects in rats (Li S.X. et al., 2018).

While NMDARs are broadly expressed in both excitatory and inhibitory neurons, the antidepressant-like effects of ketamine seem to require actions at inhibitory interneurons (iINs) (Miller et al., 2015; Widman and McMahon, 2018; Gerhard et al., 2020). As such, the “disinhibition hypothesis” posits that ketamine’s antagonism of NMDARs on GABAergic iINs causes the *dis*inhibition of downstream excitatory pyramidal neurons (PNs), resulting in activity-dependent synaptic plasticity (that requires protein synthesis) and antidepressant-like or neuroprotective effects (Miller et al., 2015; Widman and McMahon, 2018; Bartlett et al., 2020; Grieco et al., 2020). In support of this hypothesis, genetic deletions of GluN2B in iINs abolished the antidepressant effects of ketamine (Gerhard et al., 2020). Similarly, at 10 mg/kg, ketamine’s inhibition of parvalbumin-positive iINs was found to be crucial for its ability to promote plasticity in the primary visual cortex using a model of monocular deprivation (Grieco et al., 2020). Widman and McMahon (2018) also demonstrated that ketamine and NMDAR antagonists with antidepressant effects function to reduce input from iINs, ultimately enhancing the excitability of excitatory PNs. While such disinhibition might be among the most immediate triggers of antidepressant cascades, other mechanisms, including direct NMDAR antagonism on PNs, are also likely involved (Widman and McMahon, 2018).

The “direct inhibition” hypothesis, also supported by a range of preclinical studies, suggests that the *direct* inhibition of PNs through NMDAR antagonism induces a rebound homeostatic synaptic plasticity (as opposed to excitation-induced activity-dependent synaptic plasticity), which in turn causes an enhanced excitatory synaptic drive onto these neurons (Miller et al., 2015). For example, Kiselycznyk et al. (2015) showed that genetic deletion of NMDARs from iINs does not prevent a GluN2B-specific antagonist from being able to elicit an antidepressant-like response in the medial prefrontal cortex (mPFC), as measured by the FST (Miller et al., 2015). The two distinct cellular pathways described by the “disinhibition” and “direct inhibition” hypotheses may not be mutually exclusive, and the relative contributions of NMDARs in inhibitory and pyramidal neuron populations may vary by brain region and synapse types, as well developmental stage (Miller et al., 2015). Furthermore, the role of NMDARs in ketamine’s mechanism of action more generally is unclear: Zanos et al. (2016) reported that the metabolite (2*R*,6*R*)-hydroxynorketamine possesses similar antidepressant effects but with substantially lower NMDAR affinity, and instead involves and early and sustained activation of α-amino-3-hydroxy-5-methyl-4-isoxazolepropionic acid receptor (AMPARs) (Highland et al., 2021). While the NMDAR-independence of ketamine’s antidepressant actions remains an ongoing debate (Collingridge et al., 2017; Suzuki et al., 2017; Zanos et al., 2017), clinical evaluations of HNK levels have also been explored (Highland et al., 2021): one study showed that higher levels of this ketamine metabolite in plasma are associated with *less* clinical improvement and suicidal thoughts (Grunebaum et al., 2019).

*N*-methyl-D-aspartate receptors are positioned both at synaptic compartments and extrasynaptic compartments. While the precise quantitative contribution of extrasynaptic NMDAR signaling (activated by excess ambient glutamate) has been classically difficult to measure (Hardingham and Bading, 2010), extrasynaptic NMDARs are thought to constitute about 29–33% of the total NMDAR pool (at least in hippocampal dendrites) (Moldavski et al., 2020) with NMDAR subunit mobility within the membrane contributing to a shifting pool of synaptic and extrasynaptic NMDARs (Tovar and Westbrook, 2002). While synaptic NMDAR signaling plays a central role in activity-dependent plasticity and downstream transcriptional changes that ultimately mediate modifications in synapse strength, extrasynaptic NMDAR signaling is linked to the downstream excitotoxic effects secondary to glutamate spillover. The predominant working model regarding these distinct NMDAR subpopulations is that the activation of synaptic NMDARs initiates pro-survival pathways, whereas the activation of the extrasynaptic receptors activate pro-apoptotic pathways (Li S.X. et al., 2018). Kokane et al. (2017) substantiate this model, showing that the cytotoxicity of repetitive ketamine exposure in the anterior cingulate cortex of neonatal rats was linked to heightened channel activity at GluN2B. Furthermore, several studies have found that selective antagonism of GluN2B, found more predominantly in extrasynaptic pools within adult model systems (Parsons and Raymond, 2014), is sufficient to produce antidepressant-like effects, including increased levels of synaptic proteins (Li et al., 2010; Xia et al., 2010; Miller et al., 2014; Kiselycznyk et al., 2015; Li S.X. et al., 2018).

The association of synaptic receptors with neuroprotection and extrasynaptic receptors with neurotoxicity seems to shed some light on the question of how the same drug can have opposite effects at different doses. However, other experiments suggest that this understanding of the difference between NMDAR subtypes may be flawed. By measuring protein levels associated with pro-death (caspase-3) and pro-survival (phosphor [p]-CREB, p-AKT, p-ERK 1/2, BDNF) signaling pathways, Zhou et al. (2013) found that NMDAR-mediated glutamate excitotoxicity in 14-day old rat primary cortical neurons was a function of the magnitude and duration of co-activation of both types of receptors. Such findings cast doubt on the hypothesis that synaptic and extrasynaptic NMDARs play oppositional roles (Zhou et al., 2013). Chen et al. (2014) further substantiated this co-activation hypothesis: extending upon the experiments done by Zhou et al. (2013) they found that in acute brain slices of the adult rat frontal cortex, NMDAR co-activation led to an inhibition of pro-survival signaling pathways (decreased levels of p-CREB, p-ERK1/2, p-AKT) and cell death. Questions surrounding these alternative hypotheses are further complicated by the substantial differences in pharmacological properties of NMDARs between laboratory rodents and humans. Differences in subunit localization, composition and resultant patterns of NMDAR inhibition may influence downstream signaling cascades and the neuroprotective/neurotoxic balance of ketamine (Chen and Roche, 2007; Hedegaard et al., 2012).

More recent explorations of ketamine’s upstream membrane targets indicate that the full-fledged antidepressant actions of ketamine require AMPAR signaling. Zanos et al. (2016) showed that (2R,6R)-HNK, which has a relatively low binding affinity for the NMDAR and does not act as an NMDAR antagonist, was nevertheless able to exert antidepressant-like effects. Blocking AMPARs with NBQX abolished ketamine’s antidepressant effects on the FST, tail-suspension test, and learned helplessness paradigm (Autry et al., 2011; Lepack et al., 2015; Miller et al., 2015; Fukumoto et al., 2019). Another study concluded that AMPAR activation was necessary for ketamine’s activation of the mammalian target of rapamycin (mTOR) pathway, and subsequent upregulation of synaptic signaling proteins and dendritic spine formation (Li et al., 2010). Maeng et al. (2008) also showed that the antidepressant effects of ketamine (0.5, 2.5, 10 mg/kg), Ro 25-6981 (1, 3, 10 mg/kg), and MK-801 (0.05, 0.1, 0.2 mg/kg) all required AMPAR activation, and that the combination of AMPAR potentiating agents with even lower doses of NMDAR antagonists might be useful for the treatment of depression.

Studies examining the involvement of AMPAR activation in ketamine’s mechanism of action have largely focused on the antidepressant effects of ketamine; a role for AMPAR signaling in broader forms of neuroprotection has not been addressed with as much vigor. Several other membrane receptors have also been demonstrated to bind ketamine, including sigma-1 receptors, nicotinic acetylcholine receptors, voltage gated calcium channels, and HCN channels (Lavender et al., 2020). In addition, Wray et al. (2019) identified a novel mechanism by which ketamine might exert its neuroprotective effect. Using an immortalized C6 glioma cell line, this group showed that ketamine induced a translocation of Gα_s_ from lipid raft to non-raft domains, resulting in an increase in intracellular cAMP and a subsequent phosphorylation and activation of transcriptional regulator CREB, ultimately upregulating production of brain-derived neurotrophic factor (BDNF). This effect was present after subunit GluN1 knockdown, demonstrating the possibility of yet another NMDAR-independent mechanism of ketamine (Wray et al., 2019). While GluN3/GluN2 subunits display a substantial reduction in glutamate or glycine-activated currents, the actions of ketamine on GluN1 lacking NMDARs remains poorly defined (Smothers and Woodward, 2007).

## Ketamine as a Regulator of BDNF Signaling

Reductions in the expression or release of BDNF, which act on tropomyosin-related kinase B (TrkB) receptors, have been implicated as both a mediator and modulator of neuronal degeneration, and as an important cellular biomarker of the depressed state (Krishnan and Nestler, 2008; Bawari et al., 2019). Two major arms of intracellular signaling responses to subanesthetic ketamine have been explored, both of which ultimately culminate in the upregulation of BDNF. The first signaling pathway emphasizes ketamine’s activation of the mTOR pathway. BDNF release activates TrkB receptors and downstream signaling pathways which in turn upregulate synaptic signaling proteins and dendritic spine formation in brain regions such as the prefrontal cortex (Jelen et al., 2021). Blockade of mTOR signaling abolished ketamine-induced synaptogenesis and the corresponding antidepressant-like behavioral responses (Li et al., 2010). Another study showed that subanesthetic ketamine (20 mg/kg) attenuates the development of levodopa-induced dyskinesia (LID) and in a 6-hydroxydopa model of parkinsonism. This long-term anti-dyskinetic effect was associated with BDNF release in the striatum and subsequent activation of ERK1/2 and mTOR pathway signaling, and the behavioral effects correlated more with changes in synaptic morphology (Bartlett et al., 2020). Although significant basic science literature suggests that ketamine’s effects are mediated by mTOR activation, a recent clinical study found that blocking mTOR with rapamycin prior to administering ketamine did not alter the antidepressant effects of ketamine after 24 h and actually prolonged the antidepressant effects after 2 weeks (Abdallah et al., 2020). These unexpected results reflect challenges in translating the preclinical findings regarding ketamine’s modulation of mTOR to a clinical setting.

Likely nodes in intracellular signaling downstream of TrkB connecting ketamine to mTOR activation include PI3K, AKT, and GSK-3 (which is inactivated by PI3K-AKT signaling). In a rat model of combined amyloid-beta and isoflurane-induced cognitive impairment, ketamine (2.5, 5, or 10 mg/kg) rescued spatial memory deficits on the Morris Water Maze paradigm and promoted BDNF production through upregulation of the PI3K/AKT/GSK-3β pathway. Ketamine-treated rats also displayed a dose-dependent reduction in Aβ peptide accumulation (Wang et al., 2019). Another study employing GSK-3α^21A/21A^/β^9A/9A^ knockin mice, which express constitutively active GSK-3 isoforms that are resistant to ketamine inhibition, demonstrated that GSK-3 inactivation is necessary for ketamine-induced upregulation of AMPAR GluA1 subunits associated with ketamine’s antidepressant effects (Beurel et al., 2016). In contrast, Tavares et al. (2018) showed that ketamine’s ability to offer cytoprotective effects against cortisone-induced cell death occur via the activation of Akt/mTOR/S6 kinase signaling without inactivating GSK-3. Harraz et al. (2016) propose a separate potential mechanism for mTOR activation and neuroprotection, in which ketamine mediates the NO/GAPDH/Siah1 NMDAR-dependent pathway. Normally, NMDAR activation leads to the degradation of the small GTPase Rheb, which prevents mTOR activation. By blocking NMDAR activation, ketamine stabilizes Rheb, which in turn increases mTOR activation (Harraz et al., 2016).

The second intracellular pathway implicated in inducing BDNF upregulation in response to subanesthetic ketamine treatment involves the eukaryotic elongation factor 2 kinase (eEF2K) as responsible for BDNF upregulation (Monteggia et al., 2013). Autry et al. (2011) focused on eEF2K rather than mTOR and found that ketamine’s fast-acting and sustained antidepressant responses required AMPAR activation and BDNF translation through the deactivation of eEF2K. In line with these findings, one study demonstrated that memantine’s inability to inhibit the phosphorylation of eEFK2 precluded it from upregulating BDNF expression and imparting an antidepressant effect (Gideons et al., 2014). The antidepressant effects of (2R-6R)-HNK (10 mg/kg intraperitoneally or 50 μM bath application) also include eEF2 inactivation (Zanos et al., 2016; Suzuki et al., 2017) and mice with deletions of eEF2K do not display ketamine-induced antidepressant effects (Nosyreva et al., 2013).

Ketamine-induced increases in BDNF may promote synaptogenesis: one study examined the effects of genetically impaired BDNF mRNA trafficking in mouse mPFC pyramidal neurons and found that ketamine-induced synaptogenesis is dependent on the dendritic translation and release of BDNF (Liu et al., 2012). Even (2R-6R)-HNK, a ketamine metabolite with a significantly lower NMDAR affinity, requires BDNF and TrkB activation for its antidepressant effects (Fukumoto et al., 2019). Finally, Autry et al. (2011) showed that 3.0 mg/kg ketamine administered to mice with a conditional *Bdnf*-knockout failed to produce antidepressant-like effects. These findings suggest that the mTOR/BDNF pathway, possibly through an NMDAR-independent mechanism, is critical for the antidepressant-like effects of ketamine (Fukumoto et al., 2019).

## Downstream Effects of Ketamine: Inflammatory Signaling, Antioxidant Stress, Autophagy, and Apoptosis

Given ketamine’s popular use as an anesthetic for pediatric patient populations (Young et al., 2005; Soriano et al., 2010; Yan and Jiang, 2014), the vast majority of studies that have explored ketamine’s potential neurotoxic effects have applied ketamine within developmental critical windows. Ketamine is known to have neurotoxic effects at high doses especially in models of developing neurons (Dong and Anand, 2013; Kurdi et al., 2014; Mion, 2017; Pribish et al., 2020), which display greater and longer NMDAR blockade when compared to mature neurons (Jin et al., 2013; Yan and Jiang, 2014) and have relatively lower quantities of GluN2A (Paoletti et al., 2013; Szczurowska and Mares, 2013). Ketamine and other NMDAR antagonists have also been described to result in cytoplasmic vacuolization in cingulate and retrosplenial neurons (“Olney’s lesions”) at doses of 40 mg/kg, but not at 5, 10, and 20 mg/kg (Olney et al., 1989). A more contemporary neuropathological evaluation compared single and multiple doses of ketamine (up to 60 mg/kg) and MK801 and 2R-6R-HNK: only rats treated with single or multiple doses of MK-801 developed evidence of Olney’s lesions (Morris et al., 2021).

Ketamine’s ability to promote either cell survival or cell death depending on its dose was aptly illustrated by a study which found that subanesthetic ketamine (5 mg/kg) prevented widespread apoptosis caused by more anesthetic doses (20 mg/kg) via the upregulation of Activity-Dependent Neuroprotective Protein (ADNP) (Brown et al., 2015). This finding may have enormous clinical relevance, given ketamine’s popularity as an anesthetic in pediatric age groups. A review by Yan and Jiang (2014) argues that even at anesthetic doses, ketamine can have neurotoxic and/or neuroprotective effects on developing brains, depending on dose, timing, frequency, and the presence or absence of noxious stimuli. Anesthetic ketamine may exert neurotoxic effects through NMDAR blockade and subsequent compensatory NMDAR upregulation, while also serving a neuroprotective role by inhibiting inflammation-mediated toxicity associated with surgery and promoting synaptic plasticity through the release of BDNF in the hippocampus (Yan and Jiang, 2014). A significant number of preclinical studies have explored the mechanisms underlying ketamine-induced neurotoxicity in search for therapeutic agents that might protect developing neurons against the neurotoxicity of ketamine (Zuo et al., 2016; Wang Q. et al., 2017; Li Y. et al., 2018). Preclinical findings, however, are not always obviously observable clinically, and long-term neurocognitive safety assessments following anesthetic ketamine exposure in pediatric age groups have not consistently identified clinically meaningful correlates of neurotoxicity (McCann and Soriano, 2019).

The neurotoxic properties of ketamine on developing neurons may be ameliorated by harnessing the very same pathways implicated in ketamine’s neuroprotective effects. For example, one study showed that siRNA mediated inhibition of GSK-3β can prevent ketamine-induced toxicity in neural stem cell-derived neurons (Zhang et al., 2018), and another showed that the antioxidant edaravone can protect cultured primary rat cortical neurons from ketamine-induced apoptosis through activation of the PI3K/Akt signal pathway and by reducing oxidative phosphorylation (Zhang et al., 2018). Additionally, several studies have linked ketamine-induced neurotoxicity to high levels of reactive oxygen species (ROS)/malondialdehyde (MDA) generation, reduced total antioxidant capacity, and subsequently to high levels of autophagy- and apoptosis-related proteins (Bai et al., 2013; Li X. et al., 2018; Li Y. et al., 2018). However, a recent study showed that in a zebrafish model, ketamine had a protective effect and actually reduced levels of ROS *in vivo* in a concentration-dependent manner (Robinson et al., 2019).

Even at the cellular level, ketamine’s effects at different dosages can be directly oppositional. The literature on ketamine’s neuroprotective potential emphasizes the increased expression of BDNF as crucial to promoting synaptogenesis, cell survival, and *in vivo*, improvement in terms of a variety of behavioral measures. However, one recent study showed that prolonged exposure of postnatal day 7 (P7) rat pups to ketamine (20 mg/kg per dose) decreased BDNF expression and the phosphorylation of AKT and ERK and increased apoptosis in the developing rat hippocampus, which corresponded with later cognitive impairments (Meng et al., 2020).

Ketamine’s activation of mTOR appears to play a central role in its neuroprotective and antidepressant-like effects, yet some studies show that mTOR activation can have differential effects on cell health (Fan et al., 2017; Mansouri et al., 2017; Liu et al., 2019). For example, Fan et al. (2017) showed that subanesthetic ketamine injections (8 mg/kg) produced neuroprotective effects in a mouse model of Parkinson disease (PD), which corresponded with elevated expression of autophagy markers and *reduced* mTOR activation compared to untreated PD mice. These results suggest that subanesthetic ketamine may support motor and cognitive function in PD by *actually enhancing* autophagy, degrading abnormally aggregated proteins and thereby preventing neuronal apoptosis (Fan et al., 2017). Liu et al. (2019) showed that ketamine treatment of cultured rat hippocampal neurons induced a dose-dependent increases in apoptosis that was associated with a dose-dependent activation of the mTOR pathway. Inhibition of mTOR signaling by rapamycin *protected* rat hippocampal neurons from ketamine-induced injuries by reducing apoptosis, oxidative stress, and Ca^2+^ concentration (Liu et al., 2019), and co-administered rapamycin blocks ketamine’s antidepressant effects (Li et al., 2010). Notably, Liu et al. (2019) used immature fetal hippocampal neurons in their study, so their results may have been impacted by known differences in the predominance and localization of GluN2A and GluN2B-containing NMDARs in developing brains as compared to adult brains (Paoletti et al., 2013). Lastly, in attempting to assess the ability of pituitary adenylate cyclase-activating polypeptide (PACAP) to shield adult neural stem cells from the neurotoxic effects of anesthetic ketamine (50 μM, 200 μM, 400 μM, and 1 mM s-ketamine medium), Mansouri et al. (2017) also showed that ketamine’s activation of AMPA receptors leads to mTOR activation *in vitro*, which, rather than promoting neuroprotection, contributed to ER stress and apoptosis.

Ketamine’s ability to exert contradictory effects extends to proteins involved in inflammation and autophagy. In evaluating ketamine’s effectiveness as a neuroprotectant in rat hippocampal tissue in a model of TBI, Wang C.Q. et al. (2017) demonstrated that at 10 mg/kg every 24 h up to 7 days, ketamine attenuates neuroinflammation by inhibiting production of IL-6 and TNF-α and downregulates autophagy by activating the mTOR pathway. One study showed that in human lymphocytes, NMDAR activation resulted in ROS production and upregulation of NMDAR expression. MK-801, a non-competitive NMDAR antagonist, mitigated this process, suggesting that the anti-inflammatory effects of ketamine might also be mediated in part by ketamine’s regulation of non-neuronal cells (Mashkina et al., 2007). Another study found that IL-6, IL-8, and IL-1β production increased in the hippocampus after 80 mg/kg intravenous ketamine administration regardless of frequency and duration (Li et al., 2017). This study also found that TNF-α expression increased after a single administration of 80 mg/kg ketamine but decreased significantly after multiple and long-term administration (20, 40, and 80 mg/kg, 6 doses at 1 h intervals) (Hudetz and Pagel, 2010; Li et al., 2017).

## Discussion

Although there has been a substantial increase in preclinical research regarding ketamine’s neuroprotective potential over the past two decades, uncertainty persists at each mechanistic step. At the cell membrane, ketamine clearly functions as an NMDA antagonist, but whether this is required for all forms of ketamine’s ultimate neuroprotective actions is less clear. AMPAR activation, whether a response to NMDAR blockade or to some other cellular effects of ketamine, appears to play an important functional role in ketamine’s antidepressant mechanisms. Ketamine’s upregulation of BDNF, crucial for synaptogenesis and adaptive plasticity mechanisms, may occur through mTOR pathway activation, inhibition of eEF2K phosphorylation, or both. A wealth of preclinical evidence also clarifies the neurotoxic effects of ketamine, with endpoints that include increased oxidative stress autophagy and apoptosis. This apparent contradiction can likely be explained by the dose of ketamine used or by molecular differences between developing and adult brains, such as the number, strength, and composition of NMDAR-containing synapses and increases in synaptic contacts that occur during development (Nosyreva et al., 2014).

A comprehensive understanding of ketamine’s effects on intracellular signaling remains a work in progress. Even within the realm of rapid antidepressant effects, there remain controversies about NMDAR independence and the cellular sites of action (PNs vs. INs). Currently, the basic science literature on ketamine paints a mixed picture as ketamine is studied in the context of a variety of disease models, cell types, brain regions, and employing a range of administration protocols (dose, frequency, route, etc.). It is therefore necessary to undertake more systematic studies of ketamine’s long-term safety that incorporate a multidisciplinary approach to incorporate measures of wellbeing, including cell death and protein (such as BDNF) levels for *in vitro* studies, and *in vivo* behavioral assessments that go beyond the forced swim test. Since ketamine is used broadly in veterinary research and medicine, non-rodent animal subjects are also viable models for study and could be used to help bridge the gap between basic science and clinical research. In addition, some standardization among cell-culture based models would allow us to obtain a more unified perspective on ketamine’s actions. Rather than focusing solely on particular nodes in the proposed mechanistic pathways, “omics”-based approaches may offer a more unbiased appreciation of the spectrum of ketamine’s cellular effects. Finally, it will be important to continue to develop and investigate analogs of ketamine that might serve as alternatives with decreased neurotoxic potential and reduced psychotomimetic effects.

## Author Contributions

DC wrote the first draft of the manuscript. All authors contributed to manuscript revision and approved the submitted version.

## Conflict of Interest

The authors declare that the research was conducted in the absence of any commercial or financial relationships that could be construed as a potential conflict of interest.

## Publisher’s Note

All claims expressed in this article are solely those of the authors and do not necessarily represent those of their affiliated organizations, or those of the publisher, the editors and the reviewers. Any product that may be evaluated in this article, or claim that may be made by its manufacturer, is not guaranteed or endorsed by the publisher.
